# Role of Oncostatin M in Exercise-Induced Breast Cancer Prevention

**DOI:** 10.3390/cancers16152716

**Published:** 2024-07-31

**Authors:** Kara A. Negrini, Dan Lin, Dhruvil Shah, Hongke Wu, Katherine M. Wehrung, Henry J. Thompson, Tiffany Whitcomb, Kathleen M. Sturgeon

**Affiliations:** 1 Department of Comparative Medicine, College of Medicine, The Pennsylvania State University, Hershey, PA 17033, USA; twhitcomb@pennstatehealth.psu.edu; 2 Public Health Sciences, College of Medicine, The Pennsylvania State University, Hershey, PA 17033, USAsturgeon.katie@gmail.com (K.M.S.); 3 Department of Medicine, College of Medicine, The Pennsylvania State University, Hershey, PA 17033, USA; 4 Department of Horticulture and Landscape Architecture, Colorado State University, Fort Collins, CO 80523, USA; henry.thompson@colostate.edu

**Keywords:** animal model, n-methyl-n-nitrosourea, motorized treadmill, myokine, oncostatin M, breast cancer prevention

## Abstract

**Simple Summary:**

Exercise is well known to decrease the risk of breast cancer. The cellular processes that contribute to exercise-induced cancer prevention are less known. We conducted an exercise training study in rats given a chemical to induce breast cancer. Exercise increased the length of time to development of breast cancer in rats. However, when we gave the rats an antibody which binds to a certain cellular protein (oncostatin M), exercise-induced protection against breast cancer was not observed. These observations may be useful in future studies as the oncostatin M protein is produced by muscles. Thus, these results support a link between muscle use during exercise and breast cancer prevention. Future studies may examine oncostatin M and other proteins produced by exercising muscles for their potential role in breast cancer prevention.

**Abstract:**

Moderate-to-vigorous-intensity physical activity decreases the risk of breast cancer. The muscle-derived cytokine (myokine), oncostatin M (OSM), has been shown to decrease breast cancer cell proliferation. We hypothesized that OSM is involved in physical activity-induced breast cancer prevention, and that OSM antibody (Anti-OSM) administration would mitigate the effect of physical activity in a rat model of mammary carcinoma. Female Sprague Dawley rats were injected with 50 mg/kg N-methyl-N-nitrosourea to induce mammary carcinogenesis. During the 20-week study, rats were exercise trained (EX) or remained sedentary (SED). Additional groups were treated with Anti-OSM antibody (SED + Anti-OSM and EX + Anti-OSM) to explore the impact of OSM blockade on tumor latency. Exercise training consisted of treadmill acclimation and progressive increases in session duration, speed, and grade, until reaching 30 min/day, 20 m/min at 15% incline. Experimentally naïve, age-matched, female rats also completed an acute exercise test (AET) with time course blood draws to evaluate OSM plasma concentrations. Relative tumor-free survival time was significantly longer in EX animals (1.36 ± 0.39) compared to SED animals (1.00 ± 0.17; *p* = 0.009), SED + Anti-OSM animals (0.90 ± 0.23; *p* = 0.019), and EX + Anti-OSM animals (0.93 ± 0.74; *p* = 0.004). There were no significant differences in relative tumor latency between SED, SED + Anti-OSM, or EX + Anti-OSM animals. Following the AET, OSM plasma levels trended higher compared to baseline OSM levels (*p* = 0.080). In conclusion, we observed that exercise-induced delay of mammary tumor development was mitigated through Anti-OSM administration. Thus, future studies of the OSM mechanism are required to lay the groundwork for developing novel chemo-prevention strategies in women who are unable or unwilling to exercise.

## 1. Introduction

Breast cancer is the second leading cause of cancer-related death in women worldwide [1]. Despite increased identification and awareness of modifiable risk factors, the incidence of breast cancer is rising and expected to continue to increase (64% from 2011 to 2030) [2,3]. There is a wide range of risk factors associated with breast cancer [4]. The most common lifestyle risks reported are smoking, obesity, alcohol consumption, and physical inactivity [4,5,6,7]. 

Biological mechanisms underlying the association between higher physical activity levels and a lower risk of breast cancer may involve several different pathways and impact all stages of carcinogenesis (initiation, promotion, and progression) [3,8,9,10]. Systemic factors identified that modulate biologic processes include muscle-derived cytokines(myokines) [8,11,12]. Myokines with endocrine effects may cause physiologic and metabolic adaptations in other organs [13,14]. Of particular interest to this study is the role of the myokine oncostatin M (OSM). Crucially, OSM is an established myokine in humans, with acute exercise sessions inducing serum elevations in younger, older, and obese people [15,16]. 

Evidence has implicated OSM as a myokine capable of inhibiting several cancer types including melanoma, lung cancer, and breast cancer [17]. Increased cell apoptosis has been described, but the primary mechanism of inhibition caused by OSM appears to be decreased cell proliferation [18]. After OSM binds the OSM-receptor (OSM-R), downstream cascades cause transcription of CCAAT/enhance-binding protein delta (C/EBPδ) within the cell nucleus. Proliferation is minimized as C/EBPδ limits progression of the cell cycle and increases the number of non-replicating cells [19]. However, emerging evidence indicates the role of OSM in neoplasia may be tissue dependent, and have a dynamic function, changing its role based on the stage of carcinogenesis [18,20,21,22]. Contradictory research supports increased levels of OSM with tumor development, tumor progression, and metastasis in cancers such as colon cancer, pancreatic cancer, and myeloma [18,20,21,23]. Glioblastoma and cervical cancer studies have identified a feed-forward loop of OSM/OSM-R-triggered STAT3 promotion of tumorigenesis [18,23,24].

To date, no study has examined the impact of OSM peptide blockade on physical activity-induced breast cancer prevention. Developing an animal model to examine the role of myokines in the oncology space could aid the discovery of novel chemo-prevention strategies in human patients. Molecular pathways that are modulated by exercise may provide drug-able targets for women at high risk of breast cancer who cannot, or will not, take part in exercise programming as part of their clinical care. 

The overall goal of this study was to describe methodology for conducting an animal model of exercise-induced breast cancer prevention and explore the functional effect of OSM peptide blockade on the model. We examined this through experiments using young, female Sprague Dawley rats with chemically induced mammary neoplasia by N-methyl-N-nitrosourea (MNU) injection. Rats were either maintained under sedentary conditions or were exercise trained throughout this study. We hypothesized that the addition of OSM blockade, through antibody administration, would mitigate the effect of exercise on tumor latency. 

## 2. Materials and Methods

Female Sprague Dawley rats (NTac:SD; *Rattus norvegicus*) were purchased from Taconic Biosciences, Inc. (Germantown, NY, USA) in four cohorts according to birth date over the course of one year. All rats were acclimated to the facility for 72 h prior to manipulation. Rats were group housed in conventional caging on corncob bedding (Teklad Corncob 7097, Inotiv, West Lafayette, IN, USA). All rats had ad libitum access to food (Teklad Global Diet 2018, Inotiv, West Lafayette, IN, USA) and filtered municipal water via automatic watering system. The housing room had a 12:12 h light:dark cycle. The room was maintained with constant temperature (72 ± 2 °F) and humidity between 30 to 70%. All rats were determined pathogen free from: *Clostridium piliforme*, *Filobacterium rodentium*, *Mycoplasma pulmonis*, *Pneumocystis* spp., Kilham Rat Virus, Rat Theilovirus, Pneumonia Virus of Mice, Rat Coronavirus, Sialodacryoadentitis Virus, Rat Minute Virus, Rat Parvovirus, Toolan’s H1 Virus, Hantaan Virus, Lymphocytic Choriomeningitis Virus, Mouse Adenovirus I and II, Reovirus 3, Sendai Virus, ectoparasites, and enteric helminths through dirty-bedding transfer to sentinel animals. Sentinel rats were collected at the conclusion of each cohort of animals. All procedures were performed at an AAALAC-International accredited facility with approval by The Pennsylvania State University College of Medicine Institutional Animal Care and Use Committee in accordance with the *Guide for the Care and Use of Laboratory Animals* [25]. 

Rats were injected intraperitoneally (IP) with 50 mg/kg N-methyl-N-nitrosourea (MNU; Tyger Scientific Inc., Ewing Township, NJ, USA) at 22-days of age and acclimated to a 5-lane motorized treadmill (Panlab Touchscreen Treadmill, Harvard Apparatus, Holliston, MA, USA) for three days prior to randomization into experimental groups [26,27]. On day 1 of acclimation, rats were placed on a stationary treadmill for 45 min. Day 2 of acclimation allowed the rats to sit on a stationary treadmill for 15 min, followed by 5 min of belt movement at 5 m/min at 0% incline. Day 3 of acclimation included 15 min of stationary treadmill, followed by 5 min at 10 m/min at 0% incline. The shock grid was not activated during acclimation. Rats were gently tapped on their tail base with a soft brush if they sat on the inactive shock grid for more than 1 s without self-correction during days 2 and 3 of acclimation.

After treadmill acclimation and group randomization, rats in the exercise training groups underwent a two-week intensity progression until reaching 20 m/min, 15% incline, 30 min/day (Table 1) [28,29]. All exercise sessions involved a 2–3 min warm-up and cool-down period immediately before and after the exercise session, respectively. All motorized treadmill sessions were conducted during the light phase of the light cycle, in the late afternoon (2:00 p.m. EST) for consistency. Exercise training was performed 5 days per week for rats in exercise training groups until euthanasia. During all exercise training episodes rats in the sedentary groups were exposed to the vibrations and noises of the treadmill by placing them on the same table as the treadmill within their home cage. 

If rats stopped running for more than 1 s (e.g., sit on shock grid without self-correction), a soft brush was tapped on their tail base. If there was no response to the brush, or if the rat was distracted by the brush, the brush would be removed from the treadmill. The edge of the brush would then be used to make a sound on the exterior of the acrylic lane divider. 

The shock grid was turned on at 0.2–0.4 mA if the rat did not self-correct within 3 s after other forms of reinforcement were attempted. Additionally, the shock grid was turned on if the rat was repeatedly non-compliant for more than 5 s at a time. Rats would be removed (i.e., shock grid would be turned off, and the rat would not be encouraged to run) from a treadmill session if the rat stopped responding to the shock grid or if the total time spent off the treadmill was greater than 20% of the total running time (6 min). The treadmill control panel recorded shock grid contact times for each lane to ensure non-compliance parameters were consistently enforced.

Rat mammary chains were palpated three times weekly for detection of tumors starting at 4 weeks post-MNU injection. Tumor latency was defined as the time to first (primary, 1°) palpable mammary changes (hyperplasia) after MNU injection (weeks post-injection; wpi). Each rats’ tumor latency was then normalized to the average latency of SED rats from the corresponding cohort in order to control for across cohort variability. Once palpable, all tumors were measured with digital calipers three times weekly to monitor for humane endpoints. Tumor measurements were also used to further classify palpable mammary changes as hyperplasia or malignancy. Primary malignancy was defined as the first palpable mammary changes that subsequently increased in size within one week (3-caliper measurements) of detection. If the size of the palpable mammary change remained static, and non-measurable with calipers throughout the remainder of this study, it was classified as primary hyperplasia. 

**Figure 1:**
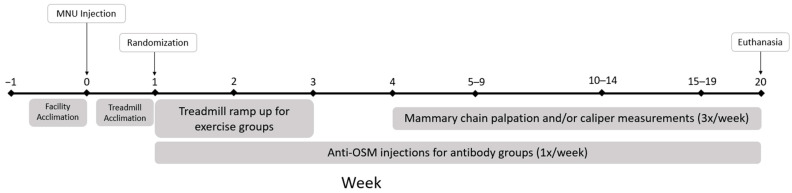
Schematic of the experimental timeline. All rats were acclimated to the treadmill prior to randomization into experimental groups. After treadmill ramp up, exercised rats continued training 5 days per week for the duration of this study (week 3–20). MNU = n-methyl-n-nitrosourea. Anti-OSM = Mouse anti-oncostatin M.

All rats survived for 20-weeks post-MNU injection, unless the rat reached a humane end point prior to experimental end point. Humane endpoints that resulted in early euthanasia were total tumor volume of 4 cm^3^ or if the location of the mammary tumor risked interfering with urination, defecation, or ambulation with growth. At 20-weeks post-MNU injection or humane endpoint, all rats were euthanized by inhalation of CO_2_ followed by exsanguination and terminal tissue collection. Heart weights and carcass weights were recorded at the time of necropsy. Rats were also assessed for additional (non-mammary) tumor development and confirmation of hyperplasia or malignancy of all mammary tumors identified. Figure 1 is a visual representation of the experimental timeline. 

Anti-OSM was administered to rats in the Anti-OSM groups at 50 µg/kg, IP once per week starting at randomization. This dose was extrapolated from administration safety pilot studies completed by our group and previous literature [30,31]. Mouse Oncostatin M/OSM Antibody (R&D Systems Inc., Minneapolis, MN, USA) was reconstituted at 33.3 μg/mL in a sterile vial using sterile 1X PBS at 7.4 pH at room temperature prior to injection. 

An additional group of age-matched, non-tumor-bearing, female Sprague Dawley rats naïve to chemical carcinogenesis and exercise training underwent a single acute exercise test (AET) on the motorized treadmill with serial blood collection. Prior to the AET, rats were acclimated to the treadmill as described. The AET consisted of a 2 min warm up period with progression to 20 m/min speed. The session lasted for 60 min, at 0% incline. Blood was collected via tail vein venipuncture before the AET, immediately after the AET, and 2 h after the AET. Blood was collected into an EDTA coated tube and then spun down to collect plasma for ELISA testing. Plasma samples were stored at −80 °C.

Plasma OSM concentrations were measured using a Rat Oncostatin M ELISA kit (Novus Biologicals, Centennial, CO, USA) per manufacturer guidelines. Blood was spun down at 10,000× *g* for 15 min at 4 °C for plasma isolation. ELISA optimization was performed and all samples were diluted 1:10 prior to testing. The ELISA absorbance reading was completed using a SPECTRAMax M2 microplate reader (Molecular Devices LLC, San Jose, CA, USA) at 450 nm wavelength absorbance profile. ELISA CV was <10% for all samples measured. 

To observe a significant difference (α level of 0.05) in tumor latency between sedentary (SED) and exercise (EX) trained groups, this study was powered at 80% with *n* = 12 per group for the primary outcome. Given considerations for animal behavior (refusal to train on the treadmill) and removal from the primary study endpoint for other reasons, we sought to randomize *n* = 30 animals. This strategy also aligned with the methodology of a 5-lane animal treadmill to maximize experimental time invested in each cohort [32]. Groups included sedentary (SED; *n* = 15) and exercise (EX; *n* = 15). One EX rat was excluded from all data analysis because of refusal to run, resulting in EX *n* = 14. No in vivo studies of OSM blockade in rats were available to power this experiment. Therefore, this cohort of animals, exercise with Anti-OSM (EX + Anti-OSM; *n* = 13), was powered similarly to EX animals to test the null hypothesis of no difference between groups (EX vs. EX + Anti-OSM). A control group of sedentary with Anti-OSM (SED + Anti-OSM; *n* = 5), was also included at a lower sample size to monitor for unexpected signal between SED and SED + Anti-OSM animals. 

All statistical analyses were performed using SAS 9.4 (Cary, NC, USA). Two-sided, unpaired t-tests were used to determine if average rat body weights were statistically significant across groups at each week. Heart weight to carcass weight ratios were compared using a one-way ANOVA with Bonferroni adjustment. Tumor latency was normalized to age-matched cohort sedentary control animals to minimize across cohort variability. Normalized latency was calculated by dividing each rats’ time to tumor palpation (in weeks) by the average latency of SED rats from their corresponding cohort. Comparisons for normalized latency and time for malignant tumor to reach 1–2 cm^3^ after palpation were assessed across all experimental groups (SED, EX, SED + Anti-OSM, EX + Anti-OSM) using a one-way ANOVA with Bonferroni adjustment. Fisher’s Exact Test was used to determine if there was a statistically different probability of a rat developing primary malignancy or primary hyperplasia. One-sided unpaired t-tests were used to determine if (1) baseline OSM concentrations were significantly lower than OSM concentrations immediately after the AET, and if (2) 2 h post-AET OSM concentrations were significantly lower than OSM levels immediately after the AET [33]. 

## 3. Results

**Figure 2:**
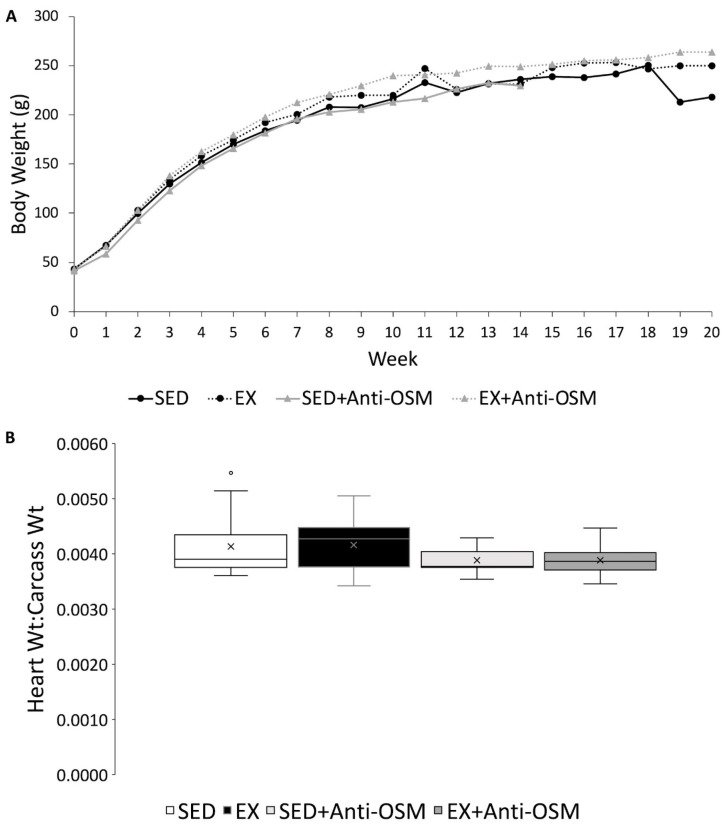
(**A**) Rat body weight growth curve over 20 weeks. Week 0 = pre-MNU injection weight. All SED + Anti-OSM rats were euthanized by week 14 due to tumor burden reaching 4 cm^3^. The drop in average SED weights during weeks 19 and 20 was due to a decrease in animal numbers from tumor burden reaching 4 cm^3^ (SED *n* = 2 at week 19). No statistical significance. (**B**) Average heart weight to carcass weight ratio. SED *n* = 15, EX *n* = 14, SED + Anti-OSM *n* = 5, EX + Anti-OSM *n* = 12. No statistical significance. x = group mean.

Rat body weights were recorded weekly with Week 0 representing the rats’ pre-MNU injection weight. Figure 2A below shows the average rat body weight growth curves for each experimental group for the duration of this study. There were no significant differences between average group body weights at any time point. The heart weight of each rat was normalized to post-mortem body weight. One rat from the EX + Anti-OSM group was excluded from the heart weight analysis due to the presence of clotted blood in the heart at necropsy. There were no significant differences noted between any groups when comparing heart-to-carcass weight ratio (Figure 2B).

**Figure 3:**
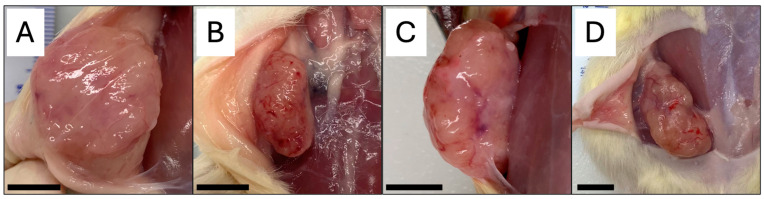
Representative photos of malignant tumors from each group. (**A**) SED. (**B**) EX. (**C**) SED + Anti-OSM. (**D**) EX + Anti-OSM. All scale bars represent 1 cm.

One EX rat was excluded from all tumor analysis due to exercise refusal after treadmill acclimation and group randomization. 91% of all rats (43 out of 47) developed palpable mammary changes over the course of this study independent of exercise or antibody administration. Four rats that did not develop palpable mammary changes were excluded from analysis (SED, *n* = 1; EX + Anti-OSM, *n* = 3). Furthermore, 35 rats (74%, *p* = 0.25) developed a primary malignant growth. On average, the amount of time for a malignant tumor to reach 1–2 cm^3^ was 15.6 ± 3.77 days for SED, 11.4 ± 2.00 days for EX, 10.6 ± 1.52 days for SED + Anti-OSM, and 11.2 ± 2.39 days for EX + Anti-OSM. Tumor growth time to 1–2 cm^3^ was not significantly different between groups. There was also no statistically significant difference in frequency of developing a primary malignant tumor compared to the frequency of primary hyperplasia (Table 2). Figure 3 shows representative images of malignant tumors on gross necropsy within each group. 

**Figure 4:**
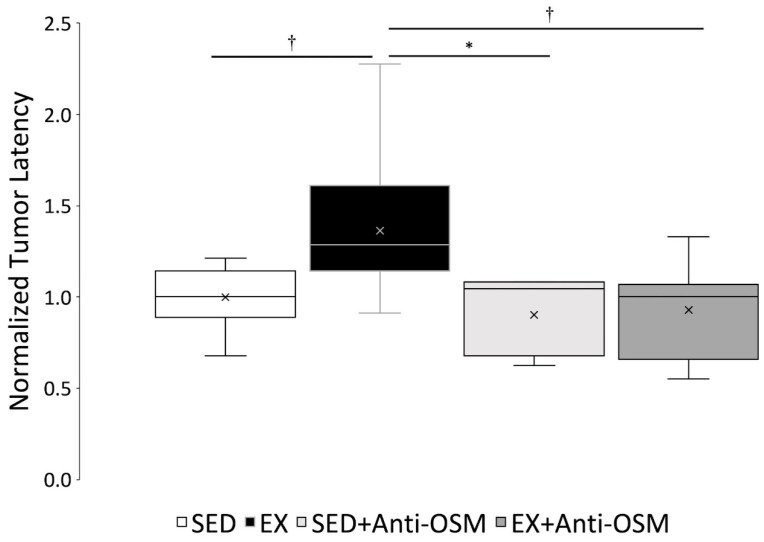
Average normalized tumor latency across groups (SED *n* = 14, EX *n* = 14, SED + Anti-OSM *n* = 5, EX + Anti-OSM *n* = 10). * *p* < 0.05, † *p* < 0.01. x = group mean.

Average tumor latency (time-to-primary hyperplasia palpation) for EX rats was prolonged (9.85 ± 3.47 wpi) compared to SED rats (8.27 ± 2.67 wpi). SED + Anti-OSM and EX + Anti-OSM rats also had a shorter tumor latency compared to EX rats (7.02 ± 1.80 and 7.79 ± 2.20 wpi, respectively). There were no significance differences observed between average tumor latency of groups. In order to control for birth cohort differences, normalized latency was calculated and compared across the four experimental groups (SED, EX, SED + Anti-OSM, and EX + Anti-OSM) to observe the effect of exercise and Anti-OSM on tumor latency (Figure 4). Average normalized latency was significantly higher for EX (1.36 ± 0.39) compared to SED (1.00 ± 0.17; *p* = 0.009) rats. EX normalized latency was also significantly higher than SED + Anti-OSM (0.90 ± 0.23; *p* = 0.019) and EX + Anti-OSM (0.93 ± 0.24; *p* = 0.004). Furthermore, there was no significant difference between SED, SED + Anti-OSM, or EX + Anti-OSM groups.

**Figure 5:**
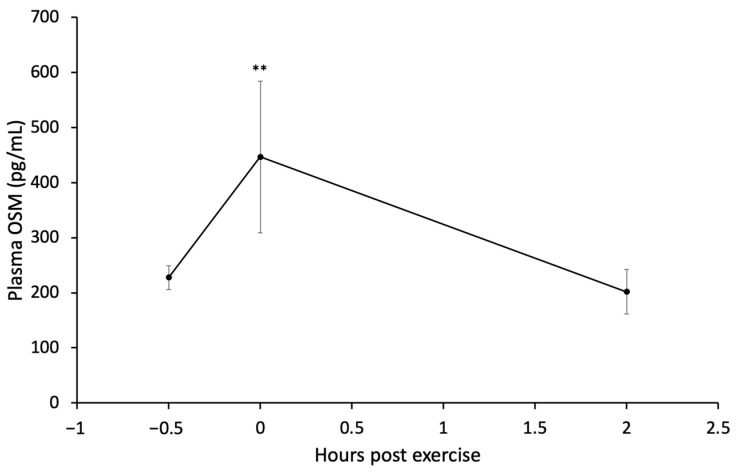
Plasma OSM concentrations after 1 h of exercise on motorized treadmill. OSM was measured from plasma samples isolated from blood collected at three different timepoints (before, immediately after, and 2 h after) from experimentally naïve rats (*n* = 7). **: *p* = 0.080.

Plasma OSM levels were obtained before, immediately after, and 2 h after rats (*n* = 7) underwent an AET to determine whether rat OSM increases in the plasma similarly to mice [33]. The average plasma OSM levels of SED, non-tumor-bearing, rats were elevated immediately to 446.8 ± 315.4 pg/mL after AET. The elevation in plasma OSM levels compared to average baseline OSM concentration (227.5 ± 52.6 pg/mL) neared significance (*p* = 0.080). Plasma OSM measured 2 h post-exercise returned to levels similar to baseline (201.9 ± 40.7 pg/mL) but were not statistically significant when comparing to immediately post-AET (*p* = 0.133) (Figure 5).

## 4. Discussion

This study is the first to report that OSM plasma levels increased in naïve Sprague Dawley rats in response to 60 min of acute exercise. It also demonstrated that exercise training delayed tumor onset in an exercise physiology-principled rat model of carcinogen-induced mammary cancer, and that blockade of OSM through administration of Anti-OSM antibody mitigated the protective effects of exercise training. 

This study utilized a validated animal model of mammary neoplasia prevention. MNU induction of mammary carcinogenesis in rats is a reliable and well-documented animal model for breast cancer research [26,34]. Doses of 50 mg/kg MNU IP in young (less than 35-day old) Sprague Dawley rats consistently induces development of mammary adenocarcinomas [26,28,35]. It has been reported that when MNU-injected rats are exercise trained, tumor onset is delayed [2,28,35,36,37]. In the present study, this dose of MNU in 22-day old, female, Sprague Dawley rats was 91% effective at causing palpable mammary changes in a 20-week time frame. In 35 out of 47 rats, these changes progressed from primary hyperplasia to primary malignancy (Table 2). Although latency of EX rats (9.85 ± 3.47 wpi) was prolonged compared to SED rats (8.27 ± 2.67 wpi), there were no significant differences across groups. Our group suspected that the increased variability in average tumor latency was secondary to having multiple birth-related cohorts of animals, and conducting behavioral (exercise training), rather than genetic or pharmacological experimental manipulation. To control for cohort variations in time-to-primary hyperplasia palpation, we explored the normalized latency of all rats. The average normalized latency of EX rats was significantly larger than SED animals (Figure 4). This supports the finding that exercise causes a delay in tumor development in our MNU breast cancer model.

Furthermore, this project sought to explore the effect of OSM blockade on tumor latency through antibody administration. A mouse Anti-OSM was utilized because rat Anti-OSM has not been validated for neutralizing the peptide for either in vitro or in vivo work. A pilot study (data not demonstrated) showed that the selected mouse Anti-OSM could be safely administered at the manufacturer’s recommended dilution in rats IP without adverse effects. Dose extrapolation combined with previous published doses in mice [31,38], led us to select a dose of 50 μg/kg IP, once weekly. Thus, we conducted a functional experiment to determine the effect of this approach [31]. At this dose, we were able to show that normalized latency for EX + Anti-OSM rats was significantly shorter compared to EX rats. EX rats also had significantly larger normalized latency compared to SED + Anti-OSM rats. There were no significant differences in normalized latency of EX + Anti-OSM rats compared to SED animals or SED + Anti-OSM rats (Figure 4). This suggests that OSM blockade in EX animals causes latencies similar to SED animals, while OSM blockade does not impact latency in rats that are sedentary. This supports our hypothesis that Anti-OSM would mitigate the effect of exercise in breast cancer prevention. 

Due to gaps in the research regarding the effect of acute exercise on OSM release by skeletal muscle in rats, an experiment was conducted to determine circulating female Sprague Dawley rat OSM levels before, immediately after, and 2 h after a single acute exercise episode. To date, this is the first report of rat plasma OSM levels. One study suggests that serum OSM increased immediately after acute exercise in mice [33]. Additional studies have observed OSM changes in humans [15,16]. We hypothesized that plasma OSM levels of rats would, similarly, elevate immediately post-exercise. We were able to observe the pattern in elevation (Figure 5). However, there was noticeable variability in the degree of plasma concentration changes for our study, indicated by a high standard deviation in the immediate post-exercise plasma OSM concentrations with only marginal significance (*p* = 0.080). 

Hojman et al. utilized *n* = 10 animals in their experimental assessment of plasma OSM levels following a 60 min swim test. We may have been underpowered at *n* = 7 for a smaller effect size from treadmill running at 0% incline [33]. Further, exercise intensity is positively associated with cancer prevention in humans as well as animal models [6,28]. We speculate that higher exercise intensities in the acute exercise test would result in higher plasma levels of OSM, and that higher intensities with exercise training could have resulted in increased tumor latency. Indeed, we did not observe cardiac adaptations to our exercise training model as indicated by the heart weight to carcass weight data (Figure 2B) [39,40]. 

There are few studies investigating the OSM pathway in reference to breast cancer prevention, in vivo [21]. Previous studies have shown the impact of OSM on breast cancer cells, in vitro [22,33]. Serum from mice immediately post-exercise caused increased caspase activity and decreased cancer cell proliferation. Exploration of gene expression revealed several possible myokines and acute factors within the serum that could have contributed to this phenomenon. The group then moved on to culture MCF-7 breast cancer cells with OSM, IL-10, IL-11, or growth differentiation factor 5 (GDF5) rich serum. OSM rich serum caused statistically significant slower cell growth compared to control cells, while the other cytokines tested did not impact cell growth [33].

Contradictory to our findings, Araujo et al. found that knocking out the OSM-R in a mouse model of mammary cancer halted tumor progression. Their study suggests that OSM expressed in the breast cancer stroma may play a role in promoting tumor progression [21]. Another in vitro study demonstrated a pro-malignant relationship between OSM-R and squamous cell carcinoma [22]. These studies demonstrate that the role of OSM may be dependent on the stage of cancer or tissue location. 

Current research utilizing Anti-OSM focuses on deriving benefits from the myokine’s overlap with the inflammatory process during both chronic inflammatory conditions and cancer [41]. Anti-OSM administration to mice has been explored as treatment options for diseases such as lupus nephritis and arthritis [30,31]. A dose of 1 mg/kg Anti-OSM IP, once weekly over a 4-week study in a mouse model of systemic lupus erythematosus, ameliorated the effects of the disease specifically related to tubulointerstitial damage of the kidney [30]. Similarly, one group found that two IP injections, separated by two days, of Anti-OSM at 100 μg/mouse may be a promising therapeutic target for rheumatoid arthritis [31]. Anti-OSM has also been explored in clinical trials of humans with rheumatoid arthritis [42]. The study showed that intravenous doses of Anti-OSM is safe for humans; however, there was limited efficacy of the antibody [42]. Our goal to interrupt the effect of OSM through administration of Anti-OSM was novel regarding mammary cancer in rats. There are no data available suggesting appropriate doses, administration schedule, or functional analysis of Anti-OSM use in rats. Thus, our results require caution as we did not conduct in vitro or other in vivo experiments to demonstrate that administration of Anti-OSM inhibits binding of OSM to OSM-R. Via our functional assay, we observed that relative tumor onset of EX rats was similar to SED rats after Anti-OSM treatment. Due to the range of literature regarding OSM involvement at different stages of breast cancer, further research would be required to identify the impact of Anti-OSM, specifically, on breast cancer at different stages of disease.

The major limitations in the present study stem from the scarcity of published information about OSM in rats. Due to the lack of information, the study design was developed to help bridge gaps required to further validate the use of Anti-OSM in rats. There are currently no studies available that offer species differences for OSM release after exercise. Hojman et al. showed that mice following a 60 min forced swim test have significantly increased serum OSM concentrations [33]. Additionally, the form of exercise could have impacted the results. Since OSM is a part of the IL-6 cytokine family and shares pathways with several inflammatory markers, it is known that OSM response may be enhanced in the face of glucocorticoid stress [41,43]. It is possible that treadmill running may elicit a different stress response compared to forced swimming due to all rats in our study being familiar with the treadmill. Therefore, the response of OSM may require different exercise parameters to consistently elicit OSM release after running on a motorized treadmill. Additional experiments would be required to determine the difference between elevation of OSM secondary to exercise or acute stressors. 

Additionally, the results were impacted by study design and execution in a few key ways. First, there was a higher-than-expected variability in the latency of all rats. MNU, while reliable at inducing mammary carcinogenesis, is not always 100% effective at inducing mammary tumors in a short time frame [26]. Since not all rats developed tumors in a 20-week period, there was increased variability in tumor latency comparisons. The natural variance associated with using an outbred stock of rats may have also impacted the birth-cohorts response to MNU. This could have been mitigated by performing the study in an inbred rat strain or changing the cohort designation. There was likely variance attributed to having multiple people performing the exercise training, while only one person was responsible for antibody administration and palpation. We attempted to remove this variability by exploring normalized data by birth cohort. 

## 5. Conclusions

To our knowledge, this is the first study demonstrating that weekly injections of Anti-OSM in rats with carcinogen-induced mammary cancer mitigates the exercise effect on tumor latency. The basis of these findings suggests that OSM release from the muscle during exercise may be an important factor in the mechanism driving exercise-induced breast cancer prevention. Future evaluations should observe circulating OSM concentrations in other animal models of exercise and cancer. Additionally, alternative dosing or timing of Anti-OSM administration should be explored to better elucidate if the protective effect of OSM depends on tumor stage. 

As the breast cancer incidence rate increases, research involving prevention strategies become increasingly more important. Rodent models will provide invaluable insight on differentiating between beneficial and detrimental effects of OSM on breast cancer. Further exploration of the impact on breast cancer initiation and progression by physical activity-induced OSM release, as well as other myokines, is critical for identifying therapeutic targets in women who are not physically active.

## Figures and Tables

**Table 1:** Motorized treadmill exercise training protocol.

Week	Day	Speed (m/min)	Grade (%)	Duration (min)
1	1	15	0	10
	2	17.5	0	15
	3	20	0	20
	4	20	5	25
	5	20	5	30
2	1	20	10	30
	2	20	10	30
	3	20	10	30
	4	20	15	30
	5	20	15	30
3+	1–5	20	15	30

**Table 2:** Frequency of tumor growth classification divided by group.

*n* (%)	SED (*n* = 15)	EX (*n* = 14)	SED + Anti-OSM (*n* = 5)	EX + Anti-OSM (*n* = 13)
Multiple Malignancies	8 (53.3)	7 (50.0)	3 (60.0)	6 (46.2)
1° Hyperplasia	5 (33.3)	1 (7.1)	1 (20.0)	1 (7.7)
1° Malignancy ^a^	9 (60.0)	13 (92.9)	4 (80.0)	9 (69.2)

^a^ *p* = 0.25; Fisher’s Exact test was used to obtain the *p* value to compare the probability of a rat developing a primary malignancy compared to primary hyperplasia.

## Data Availability

The datasets presented in this article are not readily available due to technical limitations. Requests to access the datasets should be directed to the corresponding author.
